# Quantifying accumulation characteristics of glutelin and prolamin in rice grains

**DOI:** 10.1371/journal.pone.0220139

**Published:** 2019-07-18

**Authors:** Min Huang, Jiana Chen, Fangbo Cao, Zui Tao, Tao Lei, Alin Tian, Yu Liu, Guanghui Chen, Yingbin Zou

**Affiliations:** Crop and Environment Research Center for Human Health, College of Agronomy, Hunan Agricultural University, Changsha, China; Florida Agricultural and Mechanical University, UNITED STATES

## Abstract

Glutelin and prolamin are the two major proteins in rice grains. Grain content of glutelin is considerably higher than that of prolamin in rice, but there is limited information on the factors determining the different grain contents of glutelin and prolamin. To address this knowledge gap, the present study compared final weight per grain and accumulation characteristics of glutelin and prolamin in four rice cultivars. Results showed that final glutelin weight per grain was 3.24–3.95 times higher than final prolamin weight per grain. Glutelin and prolamin accumulation processes were well fitted by the logistic equation. The initial, maximum, and mean accumulation rates of glutelin were 1.69–4.67 times higher than those of prolamin. The active accumulation duration of glutelin was 2.9–5.1 d longer than that of prolamin. These results indicate that both higher accumulation rate and longer active accumulation duration are responsible for the higher final weight per grain of glutelin compared to prolamin in rice.

## Introduction

Rice is not only the main source of calories but is also an important source of nutrition for over half of the world’s population [1]. Many rice consumers are among the world’s poorest people and have diets that are largely restricted to rice because it is filling and is the most accessible and affordable food [2]. However, rice is deficient in many nutrients [3]. Therefore, improving the nutritional value of rice is critical for improving the health of rice consumers, especially those living in poverty.

Proteins are important nutritional components in rice and it has long been recognized that glutelin and prolamin are the two major proteins in rice grains. A considerable difference exists in glutelin and prolamin contents in rice grains, i.e., 60–80% vs. 5–25% of the total protein content [4, 5, 6]. Although glutelin and prolamin have been reported to exhibit different temporal accumulation patterns during grain development in rice [6], limited information is available on the reasons for the differences in contents of glutelin and prolamin in rice grains.

Logistic regression analysis is a commonly used tool to fit the grain-filling process in rice, and parameters deduced from the regression equation (e.g. grain-filling rate and duration) are useful for understanding the factors that determine grain weight [7, 8, 9]. In this study, final weight per grain and accumulation characteristics of glutelin and prolamin were compared in four rice cultivars based on logistic regression. The main objective of this study was to determine the critical accumulation parameters contributing to the different grain contents of glutelin and prolamin in rice.

## Materials and methods

### Ethics statements

No specific permissions were required for the activities conducted in this study. The field used in this study is neither privately owned nor protected. The experiments did not involve endangered or protected species.

### Data collection

Four rice cultivars, Luliangyou 996, Xiangzaoxian 24, Xiangzaoxian 32, and Zhuliangyou 189, were grown in a field in Yongan Town (28°09′ N, 113°37′ E, 43 m asl), Hunan Province, China in the early rice-growing season in 2018. These cultivars have been widely grown by rice farmers in the study region. The soil of the experimental field was a clay with the following properties: pH 6.25, 37.3 g organic matter kg^–1^, 172 mg available N kg^–1^, 18.2 mg available P kg^–1^, and 80.7 mg available K kg^–1^. The soil test was based on samples taken from the 0–20 cm layer before the experiment began.

The cultivars were arranged in a randomized complete-block design with three replicates and a plot size of 40 m^2^. Seeds were sown according to the procedures described by Huang et al. [10]. Twenty-five-day-old seedlings were transplanted on 20 April with a high-speed rice transplanter (PZ80-25, Dongfeng Iseki Agricultural Machinery Co., Ltd., Xiangyang, China). Transplanting was done at a spacing of 25 cm × 11 cm. Missing plants were replanted by hand at 7 days after transplanting to ensure a uniform plant population. Nitrogen was applied in three splits: 67.5 kg N ha^–1^ as basal fertilizer (1 day before transplanting), 27 kg N ha^–1^ at early-tillering (7 days after transplanting), and 40.5 kg N ha^–1^ at panicle initiation. Phosphorus (67.5 kg P_2_O_5_ ha^–1^) was applied as basal fertilizer. Potassium (135 kg K_2_O ha^–1^) was split equally as basal fertilizer and at panicle initiation. The experimental field was kept flooded from transplanting until 7 days before maturity. Insects, diseases, and weeds were intensively controlled using chemicals.

About 150 main-stem panicles that headed on the same day were tagged in each plot. Seven tagged panicles were sampled randomly from each plot, starting at 3 days after full heading (when more than 80% of plants showed panicles) and then at 3 day intervals until maturity. The grains in the middle part of the sampled panicle were hand threshed, hulled, and oven-dried at 70°C to a constant weight. The hulled grains were ground into flour and passed through a 100-mesh sieve to determine glutelin and prolamin concentrations. The glutelin and prolamin in rice flour were extracted with 0.1 mol L^–1^ NaOH and 70% ethanol, respectively, according to the procedures of Agboola et al. [11]; concentrations of glutelin and prolamin were then determined by the Coomasie Blue G250 method [12]. Glutelin and prolamin weights per grain were calculated by multiplying the grain weight by the concentrations of glutelin and prolamin, respectively.

### Data analysis

Accumulation processes (i.e. changes in weight with days after full heading) of glutelin and prolamin were fitted using the logistic equation, and accumulation parameters including initial, maximum, and mean accumulation rate and active accumulation duration were calculated according to Shi et al. [8]. Data of final glutelin and prolamin weights per grain were analyzed using analysis of variance (Statistix 8, Analytical software, Tallahassee, FL, USA). Means of cultivars were compared based on the least significant difference test (LSD) at the 0.05 probability level.

## Results

**Fig 1:**
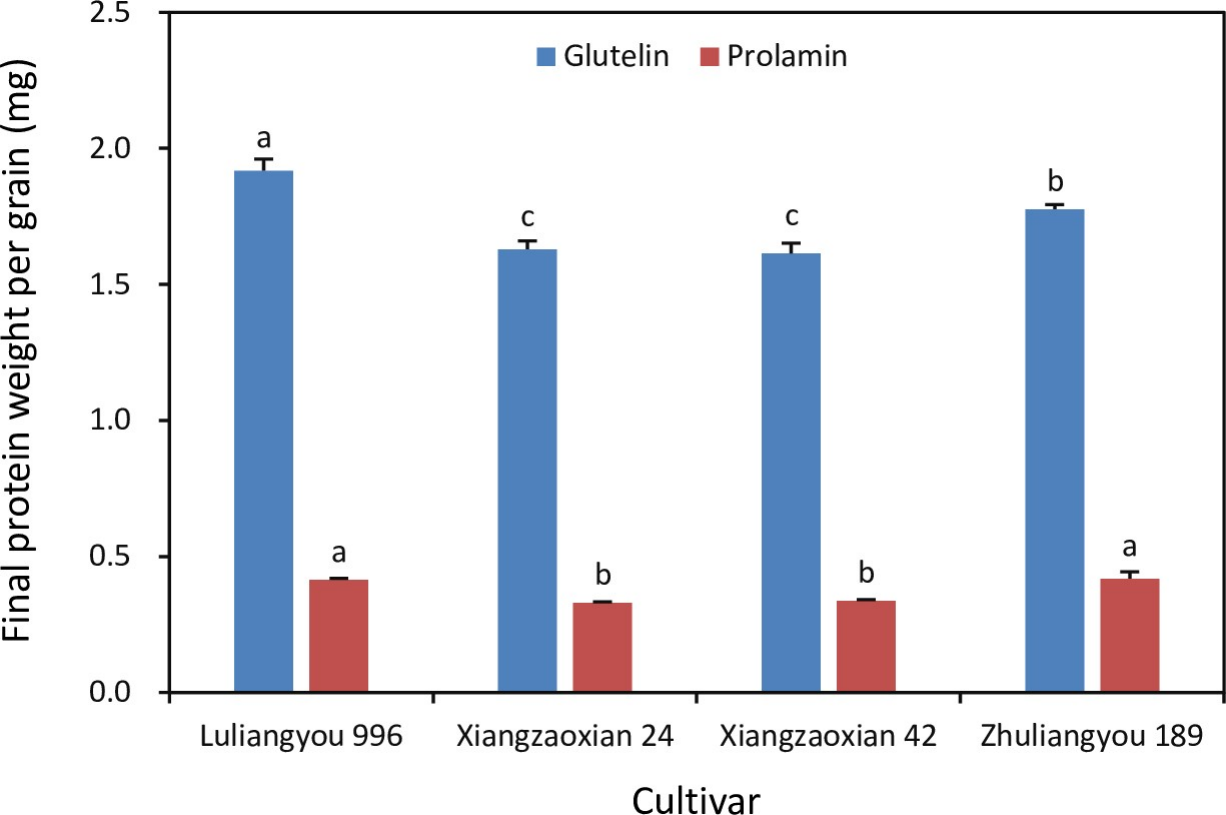
Final glutelin and prolamin weight per grain of four rice cultivars. Each column is the mean of three replicates. Error bars show standard errors. Means of each protein not sharing any letter are significantly different by the LSD test at the 0.05 probability level.

Final glutelin weight per grain was 3.24–3.95 times higher than final prolamin weight per grain in all four rice cultivars (Fig 1). The highest final glutelin weight per grain occurred in Luliangyou 996, which was 8% higher than that in Zhuliangyou 189 and about 18% higher than those in Xiangzaoxian 24 and Xiangzaoxian 42. Luliangyou 996 and Zhuliangyou 189 had similar final prolamin weights per grain, which were approximately 25% higher than those in Xiangzaoxian 24 and Xiangzaoxian 32.

**Fig 2:**
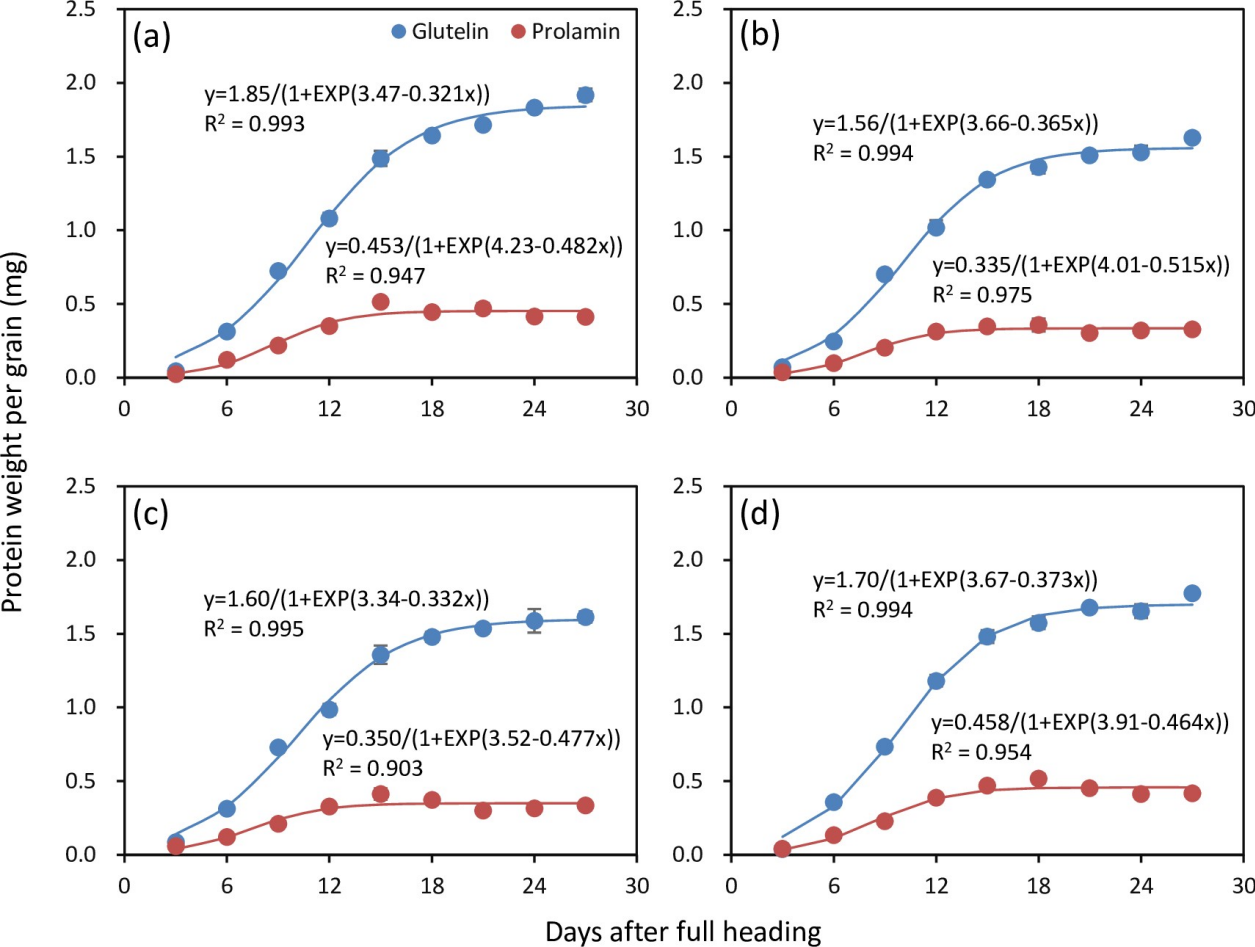
Accumulation processes of glutelin and prolamin in grains fitted by logistic equation for four rice cultivars. A, Luliangyou 996; B, Xiangzaoxian 24; C, Xiangzaoxian 42; and D, Zhuliangyou 189.

**Fig 3:**
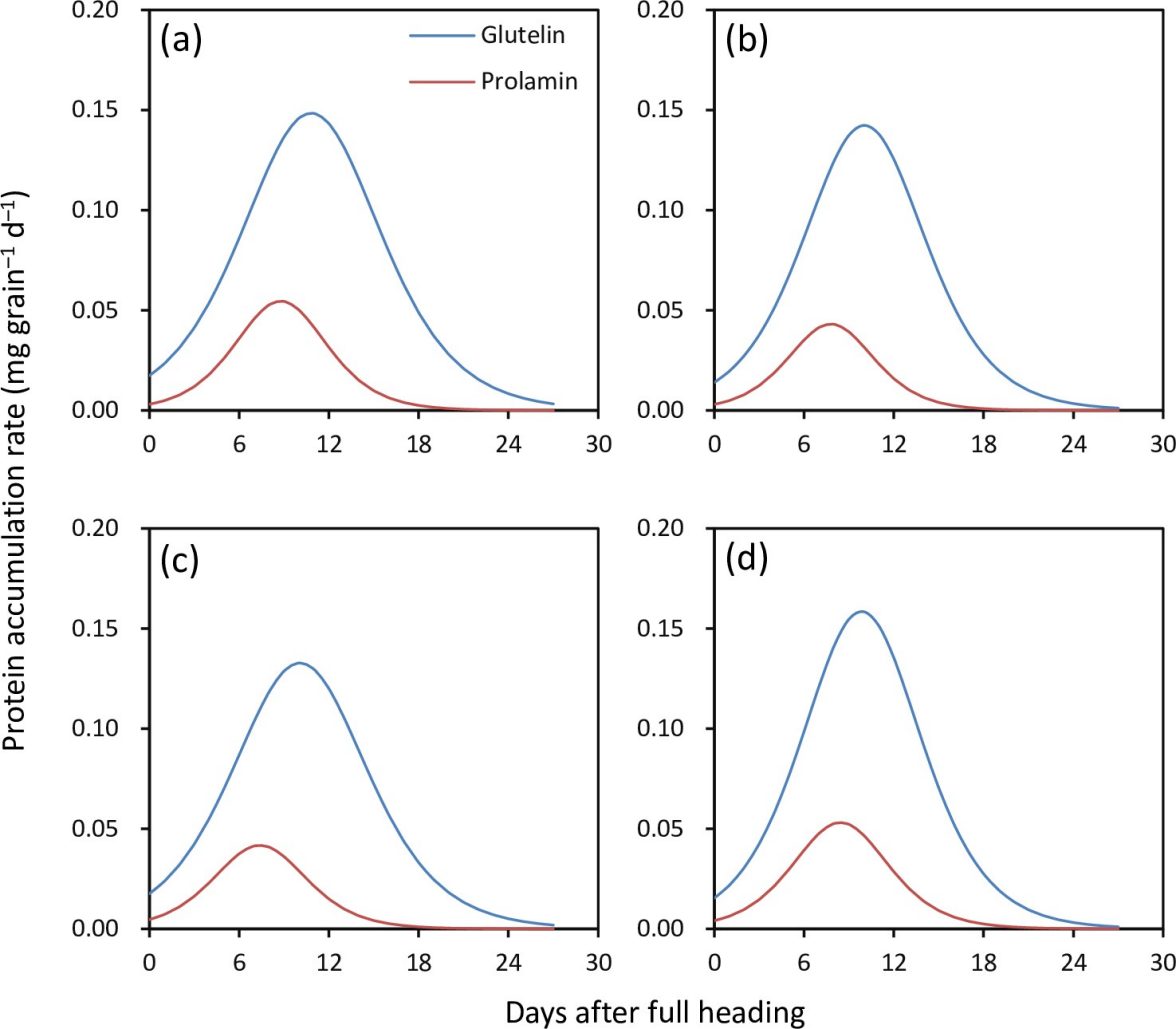
Accumulation rates of glutelin and prolamin in grains of four rice cultivars. A, Luliangyou 996; B, Xiangzaoxian 24; C, Xiangzaoxian 42; and D, Zhuliangyou 189.

Accumulation processes of glutelin and prolamin were well fitted by the logistic equation (R^2^ = 0.903–0.993) for all four rice cultivars (Fig 2A–2D). There were large differences in accumulation rates between glutelin and prolamin in all four rice cultivars (Fig 3A–3D). The initial accumulation rate of glutelin was 2.60–4.67 times higher than that of prolamin (Table 1). The maximum accumulation rate of glutelin was 1.69–2.30 times higher than that of prolamin. The mean accumulation rate of glutelin was 1.96–2.45 times higher than that of prolamin. There was a consistent difference in active accumulation duration between glutelin and prolamin across four rice cultivars; the active accumulation duration of glutelin was 2.9–5.1 d longer than that of prolamin.

**Table 1:** Accumulation characteristics of glutelin and prolamin in grains of four rice cultivars based on logistic regression.

Cultivar	Protein	Accumulation rate (mg grain^–1^ d^–1^)			Active accumulation duration (d)
		Initial	Maximum	Mean	
Luliangyou 996	Glutelin	0.017	0.148	0.074	20.0
	Prolamin	0.003	0.055	0.025	14.9
Xiangzaoxian 24	Glutelin	0.014	0.142	0.069	18.1
	Prolamin	0.003	0.043	0.020	13.5
Xiangzaoxian 42	Glutelin	0.018	0.133	0.067	18.9
	Prolamin	0.005	0.042	0.021	13.6
Zhuliangyou 189	Glutelin	0.015	0.159	0.077	17.7
	Prolamin	0.004	0.053	0.025	14.8

All accumulation parameters of glutelin and prolamin varied with cultivars (Table 1). The highest initial accumulation rate was recorded in Xiangzaoxian 42 for both glutelin and prolamin. The highest maximum accumulation rates were observed in Zhuliangyou 189 and Luliangyou 996 for glutelin and prolamin, respectively. The highest mean accumulation rate of glutelin was found in Zhuliangyou 189, while Luliangyou 996 and Zhuliangyou 189 had similar and the highest mean accumulation rates of prolamin. The longest active accumulation duration was observed in Luliangyou 996 for both glutelin and prolamin.

## Discussion

The present study showed that final glutelin weight per grain was more than 3 times higher than final prolamin weight per grain in all four tested rice cultivars. This finding is consistent with previous studies [4, 5, 6], which showed that glutelin and prolamin accounted for 60–80% and 5–25%, respectively, of the total protein in rice grains.

Until now, no modeling studies have been performed on the accumulation process of glutelin and prolamin in rice grains. In this study, we used logistic regression to fit glutelin and prolamin weight per grain onto days after full heading in four rice cultivars; a high coefficient of determination (R^2^) of more than 0.9 was obtained for all tested cultivars. This result suggests that the logistic regression is a reliable method to fit the accumulation processes of glutelin and prolamin in rice grains. In addition, the similar accumulation trends of glutelin and prolamin also indicate that protein turnover is not the basis for differences in their contents in rice grains. This finding is in agreement with Li and Okita [6].

Logistic regression analysis is a useful tool to fit the grain-filling process in rice, and parameters deduced from the regression equation (e.g. grain-filling rate and duration) have been successfully used to understand the factors that determine grain weight [7, 8, 9]. In the present study, we introduced some of the parameters and found that the higher final glutelin weight per grain compared to prolamin was attributable to both higher accumulation rate and longer active accumulation duration in rice.

Significant differences in both final glutelin and prolamin weight per grain were observed among cultivars. However, there was no consistent accumulation parameter for explaining these differences across cultivars. For example, the higher final glutelin per grain in Luliangyou 996 compared to Xiangzaoxian 24 and Xiangzaoxian 42 was attributed to both higher accumulation rate and longer active accumulation duration, while the higher final glutelin per grain in Luliangyou 996 compared to Zhuliangyou 819 was only due to longer active accumulation duration. The results of this study demonstrate that there are several possible approaches (i.e., increasing accumulation rate, prolonging accumulation duration, and both increasing accumulation rate and prolonging accumulation duration) to improve final glutelin and prolamin weight per grain in rice.

There are two limitations should be acknowledged in the present study. First, although significant differences existed in both glutelin and prolamin in grains among the tested cultivars, the variations are not wide enough to make a general conclusion. Previous studies have shown large genetic variations in total protein, glutelin and prolamin in the rice grain [13–15]. Second, this study only did a simple quantitative analysis of glutelin and prolamin accumulation in rice grains, whereas the accumulation of subunits of the proteins was not determined. Such information would be useful for improving the protein quality of rice. Therefore, further investigations are required to identify the accumulation characteristics of glutelin and prolamin and their subunits in rice grains with a more diverse set of genotypes.
